# Profile of patients with hypertension and/or *diabetes mellitus* from Primary Healthcare units

**DOI:** 10.31744/einstein_journal/2020AO4483

**Published:** 2020-01-27

**Authors:** Flavio Sarno, Clarissa Alves Gomes Bittencourt, Simone Augusta de Oliveira

**Affiliations:** 1 Instituto Israelita de Responsabilidade Social Hospital Israelita Albert Einstein São PauloSP Brazil Instituto Israelita de Responsabilidade Social, Hospital Israelita Albert Einstein, São Paulo, SP, Brazil.

**Keywords:** Chronic disease, Hypertension, Diabetes mellitus, Primary Health Care, Population characteristics

## Abstract

**Objective:**

To analyze the characteristics of patients with hypertension and/or *diabetes mellitus* from Primary Healthcare units.

**Methods:**

This is a retrospective study, with data collected from December 2014 of patients with hypertension and/or diabetes from 13 Primary Healthcare units located in the Southern region of Sao Paulo (SP, Brazil). Patients were compared by sex, diagnosis and cardiovascular risk using student *t* test, one way analysis of variance (ANOVA), and Mann-Whitney, Kruskal-Wallis and χ^2^ tests.

**Results:**

We evaluated 28,496 patients aged 20 years to 79 years (mean of 57.8 years). Most of patients were women (63.2%) and aged 50 years old or older (74.2%). The participation in the *Programa Remédio em Casa* (Medicine at Home Program) was higher among women (12.7%), and the proportions of hypertension, diabetes and both diseases were 68.0%, 7.9% and 24.1%, respectively. Patients with hypertension and diabetes had higher participation in Medicine at Home Program (13.3%), and those with diabetes only had higher participation in *Programa de Automonitoramento Glicêmico* (Self-Monitoring Glucose Program) (20.0%). The proportions of low, moderate, and high cardiovascular risk were 33.0%, 15.5%, and 51.5%, respectively.

**Conclusion:**

The sample of this study consisted of patients who were mostly women, aged 50 years or older and diagnosed with hypertension. Almost a quarter of patients also had diabetes and approximately one third and half of them were classified as low and high cardiovascular risk.

## INTRODUCTION

Arterial hypertension and *diabetes mellitus* are the two most prevalent chronic diseases worldwide. In population-based studies including individuals aged older than 18 years, global hypertension prevalence in 2015 was estimated in 24.1% for men and 20.1% for women, which represents around 1.13 billion people.^( 1 )^ Regarding diabetes, systematic review of the literature estimated these values in 8.8%, which would correspond to 415 million people aged between 20 and 79 years.^( 2 )^

In Brazil, hypertension and diabetes prevalence had similar values. In a study conducted in all Brazilian states capitals and Brazil capital in 2016, prevalence of hypertension in population aged older than 18 years was estimated in 25.7% and 8.9% in those with diabetes.^( 3 )^

Besides being prevalent, these diseases are often associated with each other. A systematic literature review reported that in most studies including patients older than 18-year-old and who had diabetes, 50% or more also had hypertension.^( 4 )^ Similarly, among patients with hypertension who were registered in following-up and recording system of arterial hypertension and *diabetes mellitus* of the Brazilian Public Health System, around 22% to 25% of them also had diabetes.^( 5 )^

In public health policy, Primary Health Care (PHC) is considered the main entrance and communication center with users in healthcare network.^( 6 )^ With regard to chronic diseases, the PHC is responsible, among other actions, for screening, diagnosis and treatment, and should also prevent, diagnosis, and treat possible complications early and coordinate the integral and continuous care of these patients.^( 7 )^

Thus, the PHC has central role in the care of patients with hypertension and diabetes. In national health survey from 2013, almost half of patients with these disease reported having their last consultation in basic health units.^( 8 , 9 )^ And between 2014 and 2015, hypertension (38.6%) and diabetes (13.6%) appeared as two most common mentioned chronic diseases by patients aged older than 18 years from PHC services in Brazil.^( 10 )^

To meet the demands and needs of these patients, knowing their characteristics is an important part of organization of working processes and care for hypertension and diabetes in PHC services.^( 11 )^

## OBJECTIVE

To analyse the characteristics of patients with hypertension and/or *diabetes mellitus* from Primary Healthcare units.

## METHODS

This is a retrospective study, with data from patients with confirmed diagnosis of hypertension and/or diabetes from 13 PHC units, located in Southern region of Sao Paulo (SP, Brazil). These PHC units are administrated by *Sociedade Beneficente Israelita Brasileira Hospital Albert Einstein,* through a partnership with the Secretary of Health of the Municipality of Sao Paulo. These patients are assisted through the Family Health Strategy, used by Brazilian Ministry of Health as model for PHC expansion and consolidation in the country.^( 6 )^

Data were obtained directly from completed spreadsheets that were used by professionals of PHC units to assist patients with hypertension and/or diabetes. The following items, comprising December 2014, were analyzed: sex, age, hypertension and/or diabetes diagnosis, cardiovascular risk (CVR), calculated according to the Framingham’s risk score, and participation of health programs (Medicine at Home Program – Medicine at Home Program and Self-Monitoring Glucose Program - SMGP). Medicine at Home Program is a home delivery of prescribed medicines to patients with stable and controlled chronic diseases, such as hypertension, diabetes, dyslipidemia, and hypothyroidism.^( 12 )^ The goal of SMGP is to provide, for insulin-dependent diabetes patients, the supplies required for self-monitoring of capillary blood glucose.^( 13 )^ Qualitative variables were described by absolute and relative frequencies, whereas age, in numerical format by mean and standard deviation. Patients were compared by sex, disease diagnosis, and CVR categories. Patients aged 20 to 79 years were inclued, once the Framingham score allows classifying CVR only in this age group. For statistical analysis, R 3.1.3 program was used, considering the significance level of 5%. Student *t* test for independent data, variance analysis (ANOVA) of one factor and Mann-Whitney, Kruskal-Wallis and the χ^2^ were used as needed.

This study was approved by Ethical and Research Committee of the *Hospital Israelita Albert Einstein* (CAAE: 46573115.6.0000.0071) and by the Secretary of Health of the Municipality of Sao Paulo (CAAE: 46573115.6.3001.0086).

## RESULTS

In December 2014, data from 28,496 patients aged between 20 and 79 years and confirmed diagnosis of hypertension and/or diabetes were analyzed. Patients’ mean age was 57.8 years, mostly women (63.2%), aged older than 50 years (74.2%), with high CVR (51.5%), and diagnosed with hypertension (68.0%). Participations in MHP and SMGP were 11.4% and 6.1%, respectively, and the former was significantly higher among women (12.7%).

We also observed statistically significant differences in the distribution of proportions between sexes and classification of CVR and diseases diagnosis ( Table 1 ).

**Table 1:** Distribution of patients, based on sex and study variables

Variables	Sex		Total	p value

	Women	Men		
Age, years	57.8±12.0	57.8±11.8	57.8±11.9	0.786^*^
Age range, years				0.797^†^
20-29	268 (1.5)	153 (1.5)	421 (1.5)	
30-39	1187 (6.6)	658 (6.3)	1.845 (6.5)	
40-49	3,200 (17.8)	1,893 (18.1)	5,093 (17.9)	
50-59	5,213 (28.9)	2,966 (28.3)	8,179 (28.7)	
60-69	5,079 (28.2)	3,099 (29.6)	8,178 (28.7)	
70-79	3,068 (17.0)	1,712 (16.3)	4,780 (16.8)	
MHP				<0.001^‡^
No	15,729 (87.3)	9,495 (90.6)	25,224 (88.6)	
Yes	2,281 (12.7)	980 (9.4)	3,261 (11.4)	
SMGP				0.510^‡^
No	16,866 (94.0)	9,789 (93.8)	26,655 (93.9)	
Yes	1,083 (6.0)	651 (6.2)	1,734 (6.1)	
CVR				<0.001^‡^
Low	5,786 (38.3)	2,064 (23.8)	7,850 (33.0)	
Moderate	2,098 (13.9)	1,584 (18.3)	3,682 (15.5)	
High	7,229 (47.8)	5,030 (58.0)	12,259 (51.5)	
Diagnosis				<0.001^‡^
Diabetes	1,231 (6.8)	1,014 (9.7)	2,245 (7.9)	
Hypertension	12,418 (69.1)	6,894 (66.0)	19,312 (68.0)	
Diabetes and Hypertension	4,323 (24.1)	2,533 (24.3)	6,856 (24.1)	
Total	18,015 (63.2)	10,481 (36.8)	28,496 (100)	

Results in means±standard deviation or n (%).* Student *t* test; ^†^ Mann-Whitney test; ^‡^ χ^2^ test.

MHP: medicine at home program; SMGP: self-monitoring glucose program; CVR: cardiovascular risk.

The proportions of patients with diagnosis of hypertension, diabetes, and both diseases (n=28,413) were 68.0%, 7.9% and 24.1%, respectively. Patients with hypertension and diabetes had a higher mean age (61.5 years) and participation in MHP (13.3%). We observed statistically significant differences in distributions of proportions between diagnosis and age and age range. Patients diagnosed with diabetes only had higher proportion of participation in SMGP (20.0%), and around one fifth of patients with hypertension (21.5%) were classified as high CVR (based on Framingham score, diabetes patients were classified with high CVR) ( Table 2 ).

**Table 2:** Distribution of patients, based on diagnosis and study variables

Variables	Diagnoses			p value

	Diabetes	Hypertension	Diabetes and hypertension	
Age, years	53.3±12.6	57.0±11.9	61.5±10.6	<0.001^*^
Age range, years				<0.001^†^
20-29	94 (4.2)	301 (1.6)	26 (0.4)	
30-39	248 (11.0)	1,380 (7.1)	213 (3.1)	
40-49	535 (23.8)	3,771 (19.5)	764 (11.1)	
50-59	656 (29.2)	5,715 (29.6)	1,782 (26.0)	
60-69	480 (21.4)	5,214 (27.0)	2,462 (35.9)	
70-79	232 (10.3)	2,931 (15.2)	1,609 (23.5)	
MHP				<0.001^‡^
No	2150 (95.8)	17,050 (88.3)	5,942 (86.7)	
Yes	94 (4.2)	2,254 (11.7)	913 (13.3)	
SMGP				<0.001^‡^
No	1,796 (80.0)	19,209 (100.0)	5,568 (81.2)	
Yes	448 (20.0)	0 (0.0)	1,286 (18.8)	
CVR				---
Low	0 (0.0)	7,842 (53.4)	0 (0.0)	
Moderate	0 (0.0)	3,680 (25.1)	0 (0.0)	
High	2,245 (100.0)	3,154 (21.5)	6,856 (100.0)	
Total	2,245 (7.9)	19,312 (68.0)	6,856 (24.1)	

Results in means±standard deviation or n (%). * ANOVA variance analysis; ^†^ Kruskal-Wallis test; ^‡^ χ^2^ test.

MHP: medicine at home program; SMGP: self-monitoring glucose program; CVR: cardiovascular risk.

In patients with calculated CVR (n=23,791), proportions of low, moderate, and high risk were 33.0%, 15.5% and 51.5%, respectively. Patients with moderate CVR had higher mean age (60.5 years). We observed statistically significant differences in the proportions distributions between CVR categories and age, age range and participation in MHP ( Table 3 ).

**Table 3:** Distribution of patients, based on cardiovascular risk and study variables

Variables	Cardiovascular risk			p value

	Low	Moderate	High	
Age, years	54.1±11.5	60.5±10.5	60.2±11.8	<0.001^*^
Age range, years				<0.001^†^
20-29	154 (2.0)	20 (0.5)	158 (1.3)	
30-39	794 (10.1)	104 (2.8)	574 (4.7)	
40-49	1,930 (24.6)	472 (12.8)	1,627 (13.3)	
50-59	2,456 (31.3)	1,076 (29.2)	3,201 (26.1)	
60-69	1,820 (23.2)	1,278 (34.7)	3,911 (31.9)	
70-79	696 (8.9)	732 (19.9)	2,788 (22.7)	
MHP				<0.001^‡^
No	6,714 (85.5)	3,144 (85.4)	10,905 (89.0)	
Yes	1,136 (14.5)	538 (14.6)	1,352 (11.0)	
SMGP				---
No	7,848 (100.0)	3,682 (100.0)	10,522 (85.9)	
Yes	0 (0.0)	0 (0.0)	1,734 (14.1)	
Total	7,850 (33.0)	3,682 (15.5)	12,259 (51.5)	

Results in means±standard deviation or n (%).* ANOVA variance analysis; ^†^ Kruskal-Wallis test; ^‡^ χ^2^ test.

MHP: medicine at home program; SMGP: self-monitoring glucose program.

## DISCUSSION

This study presented the characteristics of patients with hypertension and/or diabetes from 13 PHC units. The sample profile consisted of patients with 57.8 of mean age, most of them women, diagnosed with hypertension and classified with high CVR.

To compare the results obtained with those published in the literature, we chose to evaluate studies carried out in PHC units in south and southeast regions of Brazil with patients who had hypertension and/or diabetes. However, consideration should be given to differences in dates, locations and evaluated samples, which could have influenced patients characteristics.

Regarding the study conducted with patients with hypertensions and/or diabetes from the PHC of city of Porto Alegre (RS) in 2011, the mean age (64 years), and the proportion of women (68.3%) were higher in comparison with our results (57.8 years and 63.2%, respectively). The mean age of patients with hypertension (64 *versus* 57.0 years), diabetes (59 years *versus* 53.3 years) and those with both diseases (65.5 *versus* 61.5 years) and the proportion of women with hypertension (69.7% *versus* 64.3%), diabetes (58.1% *versus* 54.8%), and with both diagnoses (67.3% *versus* 63.1%) were also higher.^( 14 )^ It should be considered, however, that in the study from 2011 all patients were evaluated, and in our study those aged up to 79 years.

In comparing our findings with a study including patients with hypertension and/or diabetes who were assisted by the family health care teams in the city of Cambé (PR) in 2011 and 2012, the proportion of women was lower (58.3% *versus* 63.2%) and the proportion of patients with 60 years or more was similar (45.6% *versus* 45.5%).^( 15 )^ It should be taken into consideration that this study evaluated patients aged 40 years or older while our study those aged 20 to 79 years old.

In a study from 2008 conducted with patients with hypertension and/or diabetes from 3 PHC units of the city of Pelotras (RS), our results showed higher proportions of women with diabetes (54.8% *versus* 46.7%) and fewer with hypertension (64.3% *versus* 69.6%) and with both diseases (63.1% *versus* 73.0%). The proportions of patients aged 60 years or older was similar to those with diabetes (31.7 *versus* 32.0%), lower among those with hypertension (42.2% *versus* 47.0%) and higher in patients with both hypertension and diabetes (59.4% *versus* 54.6%).^( 16 )^ In these comparisons, it should be considered, again, that our study evaluated patients aged up to 79 years.

Our results showed higher participation of women in MHP, *i.e.* , 70.0% of participants of the program were women. A similar result was observed in a study conducted in the city of Rio de Janeiro (RJ) in 2005, in which women represented 71.7% of enrolled participants.^( 17 )^ Regarding to SMGP, 20.0% of patients with diabetes and 18.8% of those with hypertension and diabetes were enrolled in the program, *i.e.* , they were using insulin. These proportions are lower than those observed in study carried out in Brazil between 2006 and 2011 including adult patients with type 2 diabetes, in which 22% were using oral medications and insulin, and 13% insulin alone.^( 18 )^ However, our results presented values above of those observed in the study conducted in the city of Ribeirão Preto (SP) from 2006 to 2007, in which 11.4% of patients with diabetes were using insulin associated with oral medicines and 3.4% used insulin isolated.^( 19 )^

Regarding diseases diagnoses, our results (68.0% with hypertension, 7.9% with diabetes and 24.1% with both diseases) were similar to a study conducted in 2011 with patients assisted by PHC of the city of Porto Alegre (RS), that reported proportions of 66,5% with hypertension, 6,5% with diabetes and 27,1% with both diseases.^( 14 )^ In the study conducted in the city of Cambé (PR), 74.6% of patients had hypertension, 3.1% had diabetes and 22.3% both diseases, which are higher in relation to hypertension, lower in relation to diabetes and similar in relation to both diseases, in comparison with our results.^( 15 )^ Another study, including patients from three basic health units from the city of Pelotas (RS), observed lower proportions regarding hypertension (63%) and diabetes (2.5%), and higher proportion among patients with both diseases (31.3%).^( 16 )^

In our study almost one third of patients were classified with low CVR and half of them with high CVR. These results are different from those found in a study conducted in the city of Cambé (PR), in which patients with hypertension and/or diabetes were classified with low (40.8%), moderate (40.2%) and high (19.0%) CVR.^( 15 )^ However, our results are similar to the study conducted with patients with hypertension with 20 or more years of age from a PHC unit in the city of Ribeirão Preto (SP), where 34.8% of patients had low CVR, 20.4% moderate CVR, and 45.0% high CVR.^( 20 )^In this study, we must consider that, although 24.1% of patients with hypertension were also diagnosed with diabetes, patients with diabetes only were not evaluated.

Within the strategic action plan for coping with chronic non-transmissible diseases in Brazil, the PHC has the role, among other aspects, of providing assistance and integral care, and organizing reference and counter reference of these patients in health care network.^( 21 )^

Regarding the strategies used by PHC in the approach, assistance and patients care with chronic diseases several types were identified. A systematic review of published results between 2006 and 2014 evaluated these interventions, using as reference elements from care model for patients with chronic diseases. Most of these studies evaluated patient-related outcome categories, using one or two elements of the model, most of the investigated diseases were hypertension and diabetes.^( 22 )^

In chronic diseases care model that is proposed by Brazilian Public Health System, the characterization of individuals, according to age, sex, hereditary factors and other risk factors, such as hypertension and diabetes, appears from the third level of risk stratum of health condition.^( 23 )^ In relation to hypertension, the use Framingham CVR score is recommended for risk stratification and follow-up of these patients.^( 24 )^ For patients with diabetes, there are recommendations to consider risk stratification according to metabolic and pressure control, hospitalizations for acute complications, presence of chronic complications, other comorbidities and social risk.^( 25 )^

Thus, patients’ stratification based on risk can be considered part of health services organization strategy to chronic diseases approach, seeking to identify patients’ different needs and to plan adequate activities based on stratum risk from each group and service resources.^( 23 )^

## CONCLUSION

Prevalence, continuous care and possible complications associated with hypertension and diabetes represents a challenge for care of these diseases. The knowledge of characteristics of patients with hypertension and/or diabetes, especially the cardiovascular risk stratification, can help in organizing the approach, assistance and care for these patients.
