# Supplementary material for: Profile of patients with hypertension and/or diabetes mellitus from Primary Healthcare units
Source: Einstein (Sao Paulo). 2020 Jan 27;18:eAO4483. doi: 10.31744/einstein_journal/2020AO4483 (PMC6986880; doi:10.31744/einstein_journal/2020AO4483)
Supplement: Translation

# Translation: Perfil de pacientes com hipertensão arterial e/ou *diabetes mellitus* de unidades de Atenção Primária à Saúde

**Authors:** Flavio Sarno, Clarissa Alves Gomes Bittencourt, Simone Augusta de Oliveira

**Affiliations:** 1 Brasil Instituto Israelita de Responsabilidade Social, Hospital Israelita Albert Einstein, São Paulo, SP, Brasil.

**Keywords:** Doença crônica, Hipertensão, Diabetes mellitus, Atenção Primária à Saúde, Características da população

## Abstract

**Objetivo:**

Analisar as características de pacientes com hipertensão arterial e/ou *diabetes mellitus* de unidades de Atenção Primária à Saúde.

**Métodos:**

Estudo retrospectivo, com dados de dezembro de 2014 de pacientes com hipertensão e/ou diabetes, de 13 unidades de Atenção Primária à Saúde localizadas na região sul da cidade de São Paulo (SP). Os pacientes foram comparados por sexo, diagnósticos e risco cardiovascular, por meio dos testes *t* de Student, análise de variância de um fator, Mann-Whitney, Kruskal-Wallis e χ^2^.

**Resultados:**

Foram avaliados 28.496 pacientes, de 20 anos a 79 anos de idade (média de 57,8 anos), sendo a maioria do sexo feminino (63,2%) e com 50 anos ou mais de idade (74,2%). A participação no Programa Remédio em Casa foi maior no sexo feminino (12,7%) e as proporções de hipertensão, diabetes e de ambas as doenças foram de 68,0%, 7,9% e 24,1%, respectivamente. Os pacientes com hipertensão e diabetes apresentaram maior proporção de participação no Programa Remédio em Casa (13,3%) e aqueles com apenas diabetes apresentaram maior proporção de participação no Programa de Automonitoramento Glicêmico (20,0%). As proporções de risco cardiovascular baixo, moderado e alto foram de 33,0%, 15,5% e 51,5%, respectivamente.

**Conclusão:**

A amostra deste estudo foi constituída por pacientes em sua maioria do sexo feminino, com 50 anos ou mais de idade e diagnóstico de hipertensão. Cerca de um quarto dos pacientes apresentava também diabetes e aproximadamente um terço e metade deles foram categorizados como risco cardiovascular baixo e alto, respectivamente.

## INTRODUÇÃO

A hipertensão arterial e o *diabetes mellitus* são duas das doenças crônicas mais prevalentes do mundo. Nos estudos de base populacional em pessoas acima de 18 anos, a prevalência global de hipertensão em 2015 foi estimada em 24,1% em homens e em 20,1% em mulheres, representando cerca de 1,13 bilhão de pessoas.^1^ Em relação ao diabetes, revisão sistemática da literatura estimou estes valores em 8,8%, o que corresponderia a 415 milhões de pessoas com idades entre 20 e 79 anos.^2^

No Brasil, as prevalências de hipertensão e diabetes apresentaram valores semelhantes. Em pesquisa realizada na capital de todos os Estados e no Distrito Federal em 2016, a prevalência de hipertensão na população acima de 18 anos foi estimada em 25,7% e a de diabetes em 8,9%.^3^

Além de prevalentes, estas doenças estão frequentemente associadas. Revisão sistemática da literatura mostrou que na maioria dos estudos com pacientes acima de 18 anos de idade e portadores de diabetes, 50% ou mais também apresentavam hipertensão.^4^ Da mesma forma, entre pacientes com hipertensão registrados no sistema de cadastramento e acompanhamento da hipertensão arterial e *diabetes mellitus* do Sistema Único de Saúde (SUS), cerca de 22% a 25% apresentavam também diabetes.^5^

Na política de saúde pública, a Atenção Primária à Saúde (APS) é tida como a porta principal de entrada e centro de comunicação de usuários na rede de atenção à saúde.^6^ No que se refere às doenças crônicas, a APS é responsável, entre outras ações, pelo rastreamento, diagnóstico e tratamento, devendo também prevenir, diagnosticar e tratar precocemente as possíveis complicações e coordenar o cuidado integral e contínuo destes pacientes.^7^

Dessa forma, a APS tem papel importante na assistência de pacientes com hipertensão e diabetes. Na pesquisa nacional de saúde de 2013, cerca de metade dos pacientes com estas doenças referiram ter realizado suas últimas consultas em Unidades Básicas de Saúde.^8 , 9^ E entre 2014 e 2015, a hipertensão (38,6%) e o diabetes (13,6%) apareceram como duas das doenças crônicas mais referidas pelos pacientes com 18 anos ou mais dos serviços de APS do Brasil.^10^

Para suprir as demandas e necessidades destes pacientes, conhecer suas características é parte importante da organização dos processos de trabalho e da assistência da hipertensão e do diabetes nos serviços de APS.^11^

## OBJETIVO

Descrever as características de pacientes com hipertensão arterial e/ou *diabetes mellitus* de unidades de Atenção Primária à Saúde.

## MÉTODOS

Trata-se de estudo retrospectivo, com dados de pacientes com diagnóstico confirmado de hipertensão e/ou de diabetes de 13 unidades de APS, localizadas na região sul da cidade de São Paulo (SP), administradas pela parceria entre a Sociedade Beneficente Israelita Brasileira Hospital Albert Einstein e a Secretaria Municipal de Saúde de São Paulo. A assistência a esses pacientes é realizada por meio da Estratégia Saúde da Família, utilizada pelo Ministério da Saúde como modelo para expansão e consolidação da APS no país.^6^

Os dados foram obtidos diretamente das planilhas preenchidas e utilizadas pelos profissionais das unidades de APS na assistência de pacientes com hipertensão e/ou diabetes. Os seguintes itens, referentes a dezembro de 2014, foram analisados: sexo, idade, diagnóstico de hipertensão e/ou diabetes, risco cardiovascular (RCV), calculado de acordo com o escore de risco de Framingham, e participação em programas de saúde (Programa Remédio em Casa − PRC − e de Automonitoramento Glicêmico − PAMG). O PRC consiste na entrega no domicílio de medicamentos prescritos para pacientes com doenças crônicas estáveis e controladas, como hipertensão, diabetes, dislipidemia e hipotireoidismo.^12^ O objetivo do PAMG é fornecer, aos pacientes com diabetes que utilizam insulina, os insumos necessários para realização da automonitorização da glicemia capilar.^13^

As variáveis qualitativas foram descritas por frequências absolutas e relativas, enquanto idade, no formato numérico, por média e desvio padrão. Os pacientes foram comparados por sexo, diagnósticos das doenças e categorias de RCV. Foram incluídos pacientes de 20 a 79 anos, uma vez que o escore de Framingham permite classificar o RCV apenas deste grupo etário.

Para as análises estatísticas, foi utilizado o programa R 3.1.3, considerando o nível de significância de 5%. Os testes *t* de Student para dados independentes, análise de variância (ANOVA) de um fator, Mann-Whitney, Kruskal-Wallis e χ^2^, foram utilizados conforme a necessidade.

O estudo foi aprovado pelos Comitês de Ética em Pesquisa do Hospital Israelita Albert Einstein (CAAE: 46573115.6.0000.0071) e da Secretaria Municipal de Saúde de São Paulo (CAAE: 46573115.6.3001.0086).

## RESULTADOS

Em dezembro de 2014, foram analisados os dados de 28.496 pacientes com idades entre 20 a 79 anos e com diagnóstico confirmado de hipertensão e/ou de diabetes. A média de idade foi de 57,8 anos, sendo a maioria do sexo feminino (63,2%), com mais de 50 anos (74,2%), RCV alto (51,5%) e diagnóstico de hipertensão (68,0%). As participações no PRC e no PAMG foram de 11,4% e de 6,1%, respectivamente, sendo, no primeiro caso, significativamente maior no sexo feminino (12,7%). Observamos também diferenças estatisticamente significativas na distribuição de proporções entre os sexos e a classificação do RCV e os diagnósticos das doenças ( Tabela 1 ).

**Tabela 1:** Distribuição dos pacientes, segundo sexo e variáveis do estudo

Variáveis	Sexo		Total	Valor de p

	Feminino	Masculino		
Idade, anos	57,8±12,0	57,8±11,8	57,8±11,9	0,786^*^
Faixa etária, anos				0,797^†^
20-29	268 (1,5)	153 (1,5)	421 (1,5)	
30-39	1187 (6,6)	658 (6,3)	1.845 (6,5)	
40-49	3.200 (17,8)	1.893 (18,1)	5.093 (17,9)	
50-59	5.213 (28,9)	2.966 (28,3)	8.179 (28,7)	
60-69	5.079 (28,2)	3.099 (29,6)	8.178 (28,7)	
70-79	3.068 (17,0)	1.712 (16,3)	4.780 (16,8)	
PRC				<0,001^‡^
Não	15.729 (87,3)	9.495 (90,6)	25.224 (88,6)	
Sim	2.281 (12,7)	980 (9,4)	3.261 (11,4)	
PAMG				0,510^‡^
Não	16.866 (94,0)	9.789 (93,8)	26.655 (93,9)	
Sim	1.083 (6,0)	651 (6,2)	1.734 (6,1)	
RCV				<0,001^‡^
Baixo	5.786 (38,3)	2.064 (23,8)	7.850 (33,0)	
Moderado	2.098 (13,9)	1.584 (18,3)	3.682 (15,5)	
Alto	7.229 (47,8)	5.030 (58,0)	12.259 (51,5)	
Diagnóstico				<0,001^‡^
Diabetes	1.231 (6,8)	1.014 (9,7)	2.245 (7,9)	
Hipertensão	12.418 (69,1)	6.894 (66,0)	19.312 (68,0)	
Diabetes e Hipertensão	4.323 (24,1)	2.533 (24,3)	6.856 (24,1)	
Total	18.015 (63,2)	10.481 (36,8)	28.496 (100)	

Resultados expressos por média±desvio padrão ou n (%). * Teste *t* de Student;^†^teste de Mann-Whitney;^‡^teste do χ^2^.

PRC: programa remédio em casa; PAMG: programa de automonitoramento glicêmico; RCV: risco cardiovascular.

As proporções dos pacientes com os diagnósticos de hipertensão, diabetes e ambas as doenças (n=28.413) foram de 68,0%, 7,9% e 24,1%, respectivamente. Pacientes com hipertensão e diabetes apresentaram maior média de idade (61,5 anos) e de participação no PRC (13,3%). Observamos diferenças estatisticamente significativas na distribuição das proporções entre os diagnósticos e idade e faixa etária. Pacientes apenas com diagnóstico de diabetes apresentaram maior proporção de participação no PAMG (20,0%) e cerca de um quinto dos pacientes com hipertensão (21,5%) foram classificados como RCV alto (de acordo com o escore de Framingham utilizado, os pacientes com diabetes são classificados como RCV alto) ( Tabela 2 ).

**Tabela 2:** Distribuição dos pacientes, segundo os diagnósticos e variáveis do estudo

Variáveis	Diagnósticos			Valor de p

	Diabetes	Hipertensão	Diabetes e hipertensão	
Idade, anos	53,3±12,6	57,0±11,9	61,5±10,6	<0,001^*^
Faixa etária, anos				<0,001^†^
20-29	94 (4,2)	301 (1,6)	26 (0,4)	
30-39	248 (11,0)	1.380 (7,1)	213 (3,1)	
40-49	535 (23,8)	3.771 (19,5)	764 (11,1)	
50-59	656 (29,2)	5.715 (29,6)	1.782 (26,0)	
60-69	480 (21,4)	5.214 (27,0)	2.462 (35,9)	
70-79	232 (10,3)	2.931 (15,2)	1.609 (23,5)	
PRC				<0,001^‡^
Não	2150 (95,8)	17.050 (88,3)	5.942 (86,7)	
Sim	94 (4,2)	2.254 (11,7)	913 (13,3)	
PAMG				<0,001^‡^
Não	1.796 (80,0)	19.209 (100,0)	5.568 (81,2)	
Sim	448 (20,0)	0 (0,0)	1.286 (18,8)	
RCV				---
Baixo	0 (0,0)	7.842 (53,4)	0 (0,0)	
Moderado	0 (0,0)	3.680 (25,1)	0 (0,0)	
Alto	2.245 (100,0)	3.154 (21,5)	6.856 (100,0)	
Total	2.245 (7,9)	19.312 (68,0)	6.856 (24,1)	

Resultados expressos por média±desvio padrão ou n (%).^*^Análise de variância;^†^teste de Kruskal-Wallis;^‡^teste do χ^2^.

PRC: programa remédio em casa; PAMG: programa de automonitoramento glicêmico; RCV: risco cardiovascular.

Nos pacientes com RCV calculado (n=23.791), as proporções de risco baixo, moderado e alto foram de 33,0%, 15,5% e 51,5%, respectivamente, sendo que pacientes com RCV moderado apresentaram maior média de idade (60,5 anos). Observamos diferenças estatisticamente significativas na distribuição de proporções entre as categorias de RCV e idade, faixa etária e participação no PRC ( Tabela 3 ).

**Tabela 3:** Distribuição dos pacientes, segundo as categorias de risco cardiovascular e variáveis do estudo

Variáveis	Risco cardiovascular			Valor de p

	Baixo	Moderado	Alto	
Idade, anos	54,1±11,5	60,5±10,5	60,2±11,8	<0,001^*^
Faixa etária, anos				<0,001^†^
20-29	154 (2,0)	20 (0,5)	158 (1,3)	
30-39	794 (10,1)	104 (2,8)	574 (4,7)	
40-49	1.930 (24,6)	472 (12,8)	1.627 (13,3)	
50-59	2.456 (31,3)	1.076 (29,2)	3.201 (26,1)	
60-69	1.820 (23,2)	1.278 (34,7)	3.911 (31,9)	
70-79	696 (8,9)	732 (19,9)	2.788 (22,7)	
PRC				<0,001^‡^
Não	6.714 (85,5)	3.144 (85,4)	10.905 (89,0)	
Sim	1.136 (14,5)	538 (14,6)	1.352 (11,0)	
PAMG				---
Não	7.848 (100,0)	3.682 (100,0)	10.522 (85,9)	
Sim	0 (0,0)	0 (0,0)	1.734 (14,1)	
Total	7.850 (33,0)	3.682 (15,5)	12.259 (51,5)	

Resultados expressos por média±desvio padrão ou n (%).^*^Análise de variância;^†^teste de Kruskal-Wallis;^‡^teste do χ^2^.

PRC: programa remédio em casa; PAMG: programa de automonitoramento glicêmico.

## DISCUSSÃO

Este estudo apresentou as características de pacientes com hipertensão e/ou diabetes de 13 unidades de APS. O perfil da amostra foi constituído por pacientes com 57,8 anos de média de idade, sendo a maioria do sexo feminino, com diagnóstico de hipertensão e categorizada como RCV alto.

De forma a comparar os resultados obtidos com os da literatura, optamos por avaliar os estudos realizados em unidades de APS das Regiões Sul e Sudeste do Brasil, com pacientes com hipertensão e/ou diabetes. Entretanto, devem ser levadas em consideração as diferenças de datas, locais e amostras avaliadas, que poderiam ter influenciado nas características dos pacientes.

Em relação ao estudo realizado com pacientes com hipertensão e/ou diabetes da atenção primária da cidade de Porto Alegre (RS) em 2011, a média de idade (64 anos) e a proporção de mulheres (68,3%) foram maiores na comparação com os nossos resultados (57,8 anos e 63,2%, respectivamente). Foram também maiores as médias de idades dos pacientes com hipertensão (64 anos *versus* 57,0 anos), diabetes (59 anos *versus* 53,3 anos) e com as duas doenças (65,5 anos *versus* 61,5 anos) e as proporções de mulheres, sendo de 69,7% *versus* 64,3% para hipertensão, 58,1% *versus* 54,8% para diabetes e 67,3% *versus* 63,1% para ambos os diagnósticos.^14^ Deve-se considerar, entretanto, que no estudo de 2011 foram avaliados todos os pacientes e, no nosso estudo, aqueles com até 79 anos de idade.

Na comparação com estudo realizado com pacientes com hipertensão e/ou diabetes assistidos por equipes de saúde da família da cidade de Cambé (PR) em 2011 e 2012, a proporção do sexo feminino foi menor (58,3% *versus* 63,2%) e a proporção dos pacientes com 60 anos ou mais foi semelhante (45,6% *versus* 45,5%) em relação aos nossos resultados.^15^ Deve-se levar em consideração que, nesse estudo, foram avaliados pacientes com 40 anos ou mais de idade e, no nosso, os pacientes com idades entre 20 a 79 anos.

Em relação ao estudo realizado com pacientes com hipertensão e/ou diabetes de 3 Unidades Básicas de Saúde da cidade de Pelotas (RS) em 2008, nossos resultados foram maiores nas proporções de mulheres com diabetes (54,8% *versus* 46,7%) e menores naquelas com hipertensão (64,3% *versus* 69,6%) e com as duas doenças (63,1% *versus* 73,0%). As proporções de pacientes com 60 anos ou mais foram semelhantes naqueles com diabetes (31,7 *versus* 32,0%), menores naqueles com hipertensão (42,2% *versus* 47,0%) e maiores nos pacientes com hipertensão e diabetes (59,4% *versus* 54,6%).^16^ Nestas comparações, deve-se considerar, novamente, que nosso estudo avaliou pacientes com até 79 anos de idade.

Nossos resultados mostraram maior participação de mulheres no PRC, sendo de 70,0% a proporção do sexo feminino dentre os participantes do programa. Resultado semelhante foi observado no estudo realizado na cidade do Rio de Janeiro (RJ) em 2005, em que mulheres representaram 71,7% dos participantes cadastrados.^17^ Em relação ao PAMG, 20,0% dos pacientes com diabetes e 18,8% daqueles com hipertensão e diabetes estavam registrados no programa, ou seja, em uso de insulina. Essas proporções são menores que as observadas em pesquisa realizada no Brasil entre os anos de 2006 e 2011 com pacientes adultos e com diabetes tipo 2, sendo que 22% faziam uso de medicamentos orais e insulina e 13% de insulina isoladamente.^18^ Entretanto, nossos resultados apresentaram valores acima do observado no estudo realizado na cidade de Ribeirão Preto (SP) em 2006 e 2007, em que 11,4% dos pacientes com diabetes estavam em uso de insulina associada a medicamentos orais e 3,4% em uso de insulina isoladamente.^19^

Em relação aos diagnósticos das doenças (68,0% com hipertensão, 7,9% com diabetes e 24,1% com ambas as doenças), nossos resultados foram semelhantes aos de estudo realizado em 2011 com pacientes assistidos pela atenção primária da cidade de Porto Alegre em 2011, com proporções de 66,5%, 6,5% e 27,1%, respectivamente.^14^ No estudo realizado na cidade de Cambé, 74,6% dos pacientes apresentavam hipertensão, 3,1% de diabetes e 22,3% ambas as doenças, proporções estas maiores em relação à hipertensão, menores em relação ao diabetes e semelhantes em relação às duas doenças, na comparação com nossos resultados.^15^ Já em relação ao estudo realizado com pacientes de três unidades básicas de saúde da cidade de Pelotas, foram observadas proporções menores de hipertensão e de diabetes (63% e 2,5%) e proporção maior nos pacientes com as duas doenças (31,3%).^16^

Nosso estudo mostrou que aproximadamente um terço dos pacientes foi classificado como RCV baixo e metade como RCV alto. Esses resultados são diferentes aos encontrados no estudo realizado na cidade de Cambé, no qual 40,8%, 40,2% e 19,0% dos pacientes com hipertensão e/ou diabetes foram classificados como RCV baixo, moderado e alto, respectivamente.^15^ Entretanto, nossos resultados são semelhantes aos de estudo conduzido com pacientes com hipertensão com 20 ou mais anos de idade de uma unidade de APS da cidade de Ribeirão Preto, em que 34,8% dos pacientes apresentaram RCV baixo, 20,4% moderado e 45,0% alto.^20^ Neste estudo, deve-se considerar que, apesar de 24,1% dos pacientes com hipertensão também apresentarem o diagnóstico de diabetes, não foram avaliados os pacientes com diagnóstico apenas de diabetes.

Dentro do plano de ações estratégicas para o enfrentamento das doenças crônicas não transmissíveis do Brasil, a APS tem o papel, entre outros, de realizar a assistência e o cuidado integral e organizar a referência e a contrarreferência destes pacientes na rede de atenção à saúde.^21^

Em relação às estratégias utilizadas pela APS na abordagem, assistência e cuidado dos pacientes com doenças crônicas, foram vários os tipos identificados. Revisão sistemática dos estudos publicados entre 2006 e 2014 avaliou estas intervenções, utilizando como referência os elementos do modelo de cuidado para pacientes com doenças crônicas. A maioria destes estudos avaliou categorias de desfechos relacionados aos pacientes, utilizando um ou dois dos elementos do modelo, e as doenças mais estudadas foram a hipertensão e o diabetes.^22^

No modelo de atenção às doenças crônicas proposto para o SUS, a caracterização dos indivíduos, segundo idade, sexo, fatores hereditários e outros fatores de risco, como hipertensão e diabetes, aparece a partir do terceiro nível de estrato de risco da condição de saúde.^23^ Em relação à hipertensão, a utilização do escore de RCV de Framingham é o que tem sido recomendado para a estratificação de risco e acompanhamento destes pacientes.^24^ Para os pacientes com diabetes, recomenda-se considerar no acompanhamento a estratificação de risco de acordo com o controle metabólico e pressórico, internações por complicações agudas, presença de complicações crônicas, outras comorbidades e o risco social.^25^

Assim, a estratificação de pacientes, segundo o risco, pode ser considerada parte da estratégia de organização dos serviços de saúde para a abordagem das doenças crônicas, procurando-se identificar as diferentes necessidades dos pacientes e planejar as atividades adequadas, conforme o estrato de risco de cada grupo e os recursos dos serviços.^23^

## CONCLUSÃO

As prevalências, os cuidados contínuos e as possíveis complicações associadas à hipertensão e ao diabetes tornam a assistência destas doenças um desafio. O conhecimento das características dos pacientes com hipertensão e/ou diabetes, em especial da estratificação do risco cardiovascular, pode ajudar na organização da abordagem, na assistência e no cuidado destes pacientes.
